# A Systematic Review on Participant Diversity in Clinical Trials—Have We Made Progress for the Management of Obesity and Its Metabolic Sequelae in Diet, Drug, and Surgical Trials

**DOI:** 10.1007/s40615-022-01487-0

**Published:** 2022-12-19

**Authors:** Crystal N. Johnson-Mann, Julie S. Cupka, Alexandra Ro, Andrea E. Davidson, Brooke A. Armfield, Frank Miralles, Asena Markal, Kiara E. Fierman, Victoria Hough, Mackenzie Newsom, Isha Verma, Abdul-Vehab Dozic, Azra Bihorac

**Affiliations:** 1 Intelligent Critical Care Center, University of Florida, 1600 SW Archer Rd, PO Box 100109, Gainesville, FL 32610-0109 USA; 2 Division of Gastrointestinal Surgery, Department of Surgery, College of Medicine, University of Florida, 1600 SW Archer Rd, PO Box 100109, Gainesville, FL 32610-0109 USA; 3 Division of Nephrology, Hypertension and Renal Transplantation, Department of Medicine, College of Medicine, University of Florida, Gainesville, FL USA; 4 College of Medicine, University of Florida, Gainesville, FL USA

**Keywords:** Obesity, Demographics, Clinical trial, Systematic review

## Abstract

**Objective:**

Individuals from Black and Hispanic backgrounds represent a minority of the overall US population, yet are the populations most affected by the disease of obesity and its comorbid conditions. Black and Hispanic individuals remain underrepresented among participants in obesity clinical trials, despite the mandate by the National Institutes of Health (NIH) Revitalization Act of 1993. This systematic review evaluates the racial, ethnic, and gender diversity of clinical trials focused on obesity at a national level.

**Methods:**

Following Preferred Reporting Items for Systematic Reviews and Meta-Analyses (PRISMA) guidelines, a systematic review of clinicaltrials.gov, PubMed, Cochrane Central, and Web of Science was undertaken to locate phase 3 and phase 4 clinical trials on the topic of obesity that met associated inclusion/exclusion criteria. Ultimately, 18 studies were included for review.

**Results:**

White non-Hispanic individuals represented the majority of clinical trial participants, as did females. No study classified participants by gender identity. Reporting of race/ethnicity was not uniform, with noted variability among racial/ethnic subgroups.

**Conclusions:**

Our findings suggest that disparities remain in the diverse racial, ethnic, and gender representation of participants engaged in clinical trials on obesity relative to the prevalence of obesity in underrepresented populations. Commitment to inclusive and intentional recruiting practices is needed to increase the representation of underrepresented groups, thus increasing the generalizability of future research.

**Supplementary Information:**

The online version contains supplementary material available at 10.1007/s40615-022-01487-0.

## Introduction

The etiology of obesity is complex and multifactorial, yet this disease process has rapidly become the leading cause of morbidity and mortality in the USA. Over the past decade, the prevalence of obesity among adults increased from 33.7 to 42.4%, and the prevalence of severe obesity increased from 5.7 to 9.2% [1, 2]. This increased prevalence is most notable among African Americans and Hispanic Americans, in whom obesity continues to surpass that of White Americans at 49.6 and 44.8% compared to 42.4%, respectively [2]. African American women have a significantly higher prevalence of obesity (56.9%) compared to other racial/ethnic groups, and African Americans overall have a significantly higher prevalence of severe obesity at 13.8% compared to other groups [2].

Black and Hispanic Americans comprise 13 and 18% of the population, respectively, yet are the populations where obesity is most prevalent and are disproportionately affected by the associated comorbid conditions [3]. These populations are grossly underrepresented as participants in clinical trials [4–15], despite the NIH Revitalization Act of 1993, which required the inclusion of racial/ethnic minority groups and women in federally funded human subject research studies [16]. A few studies (overweight and obesity trials) achieved very high percentages of participation from underrepresented groups, but this is a result of pursuing recruitment in areas of majority and minority populations [17–20]. Sex differences in studies also differ, with females being significantly overrepresented as participants in weight-loss and bariatric surgery studies [21–26]. This paucity of sex and racial/ethnic diversity in clinical research can lead to research findings that are not generalizable to all members of society, with negative downstream effects for underrepresented populations most affected by the comorbid conditions associated with obesity.

The purpose of this systematic review is to examine and evaluate the racial, ethnic, and gender representation of participants in clinical trials focused on the management of obesity and its metabolic sequelae at a national level in the diet, drug, and surgical trials.

## Methods

### Search Strategy and Data Extraction

We followed the 2020 PRISMA guidelines attached as Appendix S1 [27]. We initially searched ClinicalTrials.gov on February 19, 2021, to assess the state of phase 3 and phase 4 clinical trials related to obesity, finding 248 trials, which we narrowed down to 95 studies of interest. Phases 3 and 4 were specifically chosen to include treatments that were proven effective in a small subset of the population and were now being tested in a diverse population. During phases 3 and 4 of clinical trials, participants should be chosen to represent the diversity of the whole population. Of those trials, only 24 had linked published results. This group of studies formed the basis for our search criteria for the remaining databases of PubMed, Cochrane Central, and Web of Science searched on July 6, 2021. The database search terms, including the initial ClinicalTrials.gov search strategy, are included in Appendix S2.

**Fig. 1:**
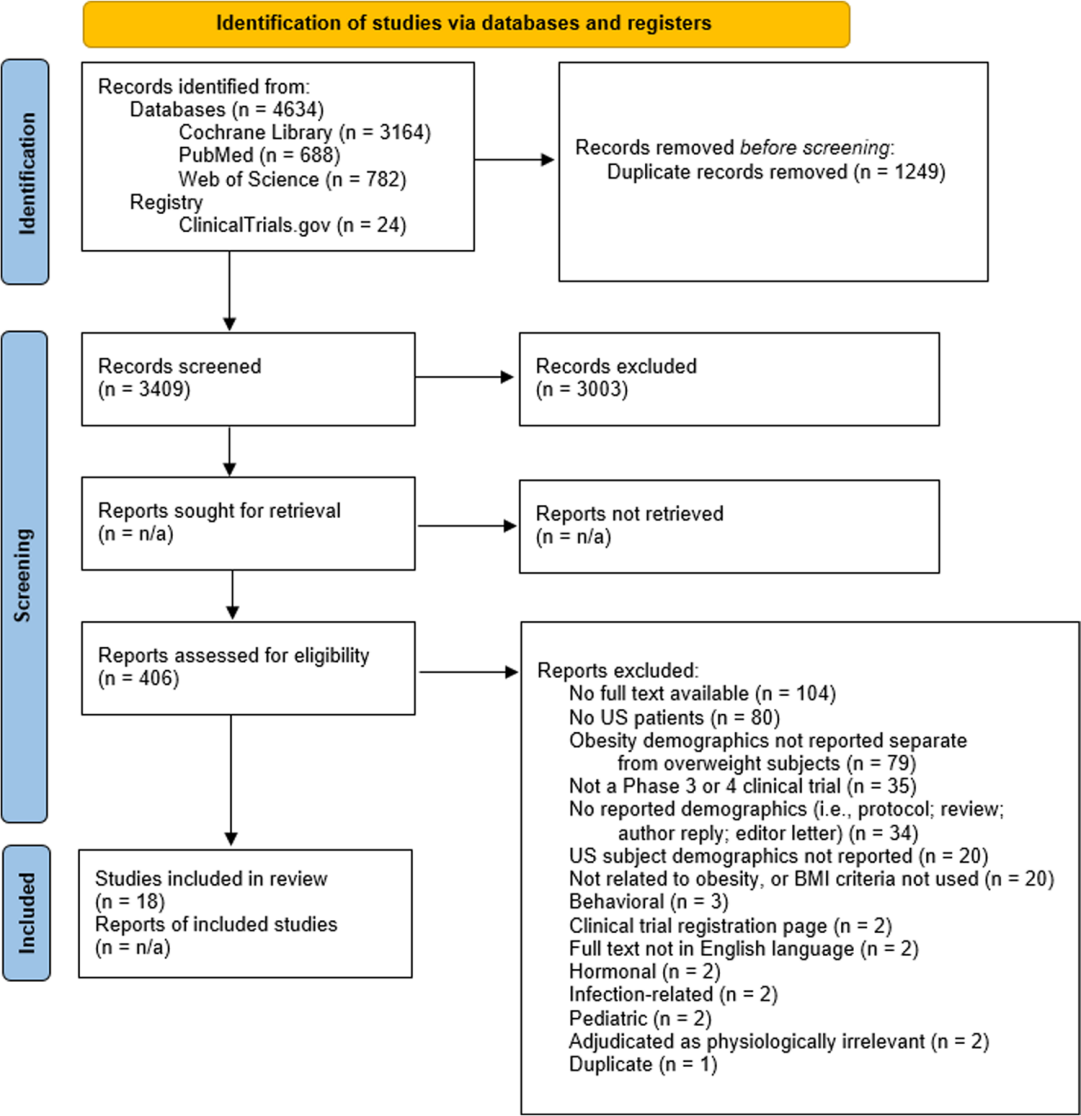
PRISMA 2020 flow diagram for new systematic reviews which include searches of databases and registers only. Adapted from Page MJ, McKenzie JE, Bossuyt PM, Boutron I, Hoffmann TC, Mulrow CD, et al. The PRISMA 2020 statement: an updated guideline for reporting systematic reviews. BMJ 2021;372:n71. https://doi.org/10.1136/bmj.n71. For more information, visit:

We retrieved 4658 articles from the database searches (Fig. 1). In total, 1249 articles were removed as duplicates leaving 3409 for the title and abstract screening. Title and abstract screening was independently performed by two reviewers, resulting in 406 articles for full-text review. During the full-text review, two independent reviewers included or excluded articles for data extraction based on the inclusion and exclusion criteria developed during the initial ClinicalTrials.gov search. Since this review is mainly concerned with treatments for obesity on adult participants within the USA, inclusion criteria were decided as: adult population (aged ≥ 18 years); reports demographics on US participants; reports demographics on subjects with obesity; phase 3 or 4 clinical trial. Studies were excluded if nonhuman, did not use body mass index (BMI) as a measure of obesity, focused on pediatrics or adolescents, or focused on the following topics: behavioral obesity; hormonal obesity; obstetric/gestational obesity; genetic obesity (i.e., Prader-Willi syndrome); infection-related obesity; psychiatric-related obesity. Any disagreements during the screening phases were brought to a consensus by a separate reviewer. This review was not registered, nor was a review protocol previously published. These limitations allow for a focused assessment of US minority patient participation in obesity-related clinical trials.

Full-text screening revealed 23 studies for data extraction, which further excluded five studies, resulting in 18 final included articles. Data extraction was independently performed by two authors, with a third reconciling extracted data when needed. Data elements included: study design details, subject demographic data, recruitment methods, whether any outcomes were related to gender/race/ethnicity, and major strengths and limitations. Demographic information was obtained from the demographic table or text of each respective article.

### Risk of Bias Assessment

We performed a risk of bias assessment on the final included articles using a worksheet adapted from both the Cochrane Review Group’s Risk of Bias Tool, version 2.0, and the methodological quality checklist of Downs and Black [28–30]. Since this review is primarily focused on demographic statistics of enrolled subjects, we focused on the risk of bias assessment on recruitment, randomization, and attrition domains. The categorical domains include allocation and randomization methods, transparency of inclusion/exclusion criteria, transparency of attrition assessments, whether enrolled patients/research staff are representative of the disease population, and transparency of recruitment methods. Two authors independently performed the risk of bias assessment, and disagreements were settled by discussion between the same two authors and a third. The risk of bias worksheet is attached as Appendix S3.

## Results

The literature search resulted in 18 included studies after de-duplication, title and abstract screening, and full-text screening. Of these 18 studies, the majority (*n* = 10) were drug trials [31–40], followed by diet trials (*n* = 7) [41–47]. One clinical trial focused on bariatric surgery [48]. Details about the trials and study population of interest are included in Table 1. Table 2 contains information on how studies defined race and ethnicity categories. Studies varied widely in how each research team categorized subjects based on demographic race and/or ethnicity information.

**Table 1:** Study characteristics including the target population, study setting, study focus, US state where the study was conducted, and the BMI criteria (in kg/m^2^) for each study included in the systematic literature review

Study	NCT ID	BMI criteria, kg/m^2^	Study focus	US states	Study setting	Targeted population
Phase 3						
[31]	NCT00563368	30–45	Drug trial	NC, PA	NR	Healthy
[32]	NCT00856609	> 30	Drug trial	AZ	Clinical research unit	Nondiabetic, otherwise healthy
[33]	NCT01508026	> 32	Drug trial	NR	NR	Stage 1 or 2 primary hypertension
[41]*	NCT00108459	≥ 30	Diet trial	PA	NR	Diabetes
[43]	NCT00696228	30–39.9	Diet trial	TN	Academic medical center	Premenopausal women
[48]*	NCT00166205	Implied**	Surgical procedure trial	NR	Academic and private practice clinical sites	Scheduled for adjustable gastric band surgery
Phase 4						
[34]	NCT02160990	≥ 30	Drug trial	MN	Clinical research unit	Accelerated gastric emptying
[35]	NCT01834404	30–40	Drug trial	MN	Clinical research unit	Healthy
[36]	NCT00791258	≥ 30	Drug trial	NR	NR	Uncontrolled hypertension on monotherapy
[37]*	NCT00339833	≥ 30	Drug trial	AZ	Clinical research unit	Nondiabetic
[38]*	NCT01035333	Implied**	Drug trial	Y	Bariatric surgery center	Scheduled for gastric bypass surgery
[39]*	NCT02833415	≥ 30	Drug trial	TX	NR	Nondiabetic
[40]*	NCT00675987	30–40 non-inclusive	Drug trial	CA, FL, IN, MA, Y, PA, TX	NR	Stage 1 hypertension, abdominal obesity, and impaired fasting glucose
[45]	NCT00079547	30–39.9	Diet trial	MO	Academic medical center	Nondiabetic
[46]*	NCT00143936	30 or higher, only included 30–40	Diet trial	CO, MO, PA	Academic medical center	Healthy
Secondary studies						
[42]*	NCT00696228	30–39.9	Diet trial	TN	Academic medical center	Premenopausal women
[44]	NCT00143936	30–40	Diet trial	COo, MO, PA	Academic medical center	Healthy
[47]*	NCT00143936	30 or higher, only included 30–40	Diet trial	CO, MO, PA	Academic medical center	Healthy

*NCT ID*, National Clinical Trials registration number; *NR*, not reported; * phase not reported in the article, but linked NCT ID reported the phase; ** BMI criteria implied due to subjects qualified for gastric band/bypass surgery

This table features the target population, study setting, study focus, US state where the study was conducted, and the BMI criteria (in kg/m^2^) for each study included in this systematic literature review

**Table 2:** Study definitions of race and/or ethnicity categories with the defined categories and relevant information pertaining to this systematic literature review

Study	Race	Ethnicity	Other	Note
[31]	Caucasian, African, Asian, other	None	American Indian, Alaskan Native, Native Hawaiian, other Pacific Islander	
[32]	NR	African American, White, Hispanic, Native American, other	Not defined*	
[33]	NR	NR	White, Black, Hispanic	
[34]	NR	Caucasian, Hispanic	NR	
[35]	NR	NR	NR	
[36]	Caucasian, Black, Asian	Hispanic/Latino	American Indian/Alaskan native	
[37]	NR	White, Native American, Hispanic	NR	
[38]	NR	NR	NR	
[39]	Black, White, other	Black, White, other	Not defined*	Study labeled groups as race/ethnicity
[40]	White, Black, other	NR	Not defined*	
[41]	White, African American, Latino, other	White, African American, Latino, other	Not defined*	Study labeled groups as race/ethnicity
[43]	Caucasian, African American	NR	NR	
[45]	NR	NR	African American	Demographics only reported in text stating that 15 of 60 subjects were African American
[46]	White, Black, Asian, American Indian or Alaska Native	Hispanic or Latino	NR	
[48]	NR	White non-Hispanic, Hispanic, Black non-Hispanic, Asian/Pacific Islander, other	Not defined*	
Sub-studies				
[42]	European American, African American	NR	NR	Self-identified with confirmation that both parents originated from the same racial group
[44]	White, African American, other	NR	Not defined*	
[47]	White, African American, Asian, other	NR	Not defined*	

*NR*, not reported; * “other” category assumed to be subjects who did not fall into another of the listed categories. This table presents the defined categories and relevant information pertaining to race and ethnicity of the included studies of this systematic literature review

**Fig. 2:**
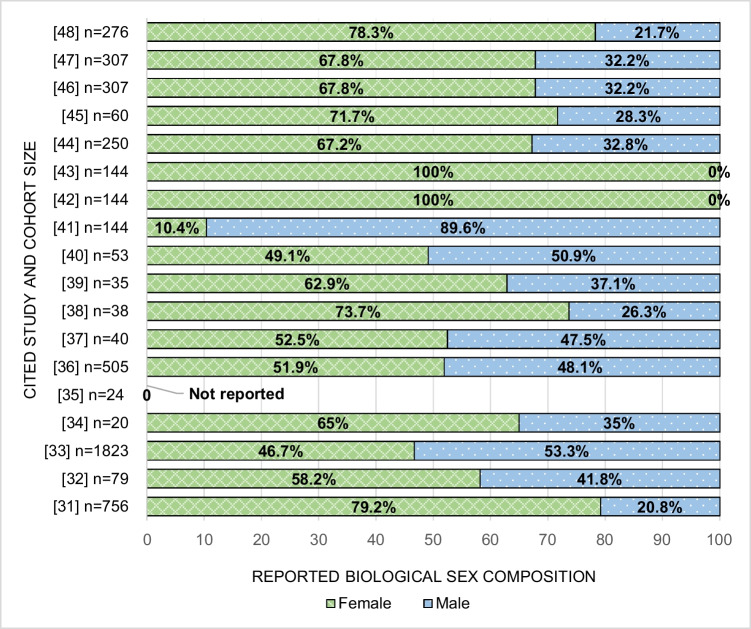
Breakdown of reported biological sex demographics for each cited study and the corresponding participant cohort size

On average, White non-Hispanic participants were the majority of participants across studies. Three studies did not report race/ethnicity as part of their cohort demographics [35, 38, 42]. Two studies had an American Indian/Alaska Native majority [32, 37], whereas no studies had an African American majority. Only one study explicitly stated whether participants could report more than one race/ethnicity [46]. Demographics by reported race/ethnicity for each study are further highlighted in Table 3. The majority of studies (*n* = 17) followed biological sex and categorized subjects as male or female [31–34, 36–48], with one not reporting sex characteristics [35]. Two studies consisted of only female participants [42, 43], and there were no male-only studies. Female participants represented the majority of subjects in 10 studies that enrolled both males and females [31, 32, 34, 38, 39, 44–48]. No study classified participants by gender identity. Figure 2 highlights the sex breakdown by study.

**Table 3:** Race and/or ethnicity demographic characteristics of subjects with obesity

								Hispanic		Non-Hispanic		Other^c^	
Study ID	Total subjects, *n* (%)	Demographic count, *n*^a^	White	AA/Black^b^	Asian	Native American or Alaskan Native	Native Hawaiian or Pacific Islander	Race	Ethnicity	Race	Ethnicity	Race	Ethnicity
Aggregate Total	4304	4915	3180 (73.9)	766 (17.8)	46 (1.1)	79 (1.8)	–	3 (0.09)	829 (19.3)	141 (3.3)	2134 (49.6)	24 (0.56)	5 (0.12)
[31]	756 (100)	762	599 (79.2)	140 (18.5)	9 (1.2)	See note	See note^c^	–	–	–	–	14 (1.9)	–
[32]	79 (100)	79	21 (26.6)	10 (12.7)	–	36 (45.6)	–	–	10 (12.7)	–	See note	–	2 (2.5)
[33]	1823 (68.3)	2429	1540 (84.5)	218 (12.0)	–	–	–	–	671 (36.8)	–	1152 (63.2)	–	–
[34]	20 (100)	20	18 (90)	–	–	–	–	–	2 (10)	–	See note	–	–
[35]	24 (100)	–	NR	NR	NR	NR	NR	NR	NR	NR	NR	NR	NR
[36]	505 (50.1)	566	329 (65.1)	139 (27.5)	31 (6.1)	6 (1.2)	–	–	61 (12.1)	–	444 (87.9)	–	–
[37]	40 (100)	40	6 (15.0)	–	–	33 (82.5)	–	–	1 (2.5)	–	39 (97.5)	–	–
[38]	38 (100)	–	NR	NR	NR	NR	NR	NR	NR	NR	NR	NR	NR
[39]	35 (100)	35	20 (57.1)	12 (34.3)	–	–	–	–	–	–	–	3 (8.6)	–
[40]	53 (100)	53	40 (75.5)	10 (18.9)	–	–	–	–	–	–	–	3 (5.7)	–
[41]	144 (100)	144	62 (43.1)	77 (53.5)	–	–	–	3 (2.1)	–	141 (97.9)	–	2 (1.4)	–
[43]	144 (100)	144	101 (70.1)	43 (29.9)	–	–	–	–	–	–	–	–	–
[45]	60 (100)	60	45 (75)	15 (25)	–	–	–	–	–	–	–	–	–
[46]	307 (100)	307	230 (74.9)	69 (22.5)	2 (0.7)	4 (1.3)	–	–	17 (5.5)	–	290 (94.5)	2 (0.7)^d^	–
[48]	276 (100)	276	169 (61.2)	33 (12.0)	4 (1.4)	–	See note^c^	–	67 (24.3)	–	209 (75.7)	–	3 (1.1)
Sub-studies													
Aggregate Total	701	557	417 (59.5)	126 (18.0)	2 (0.29)	–	–	–	–	–	–	12 (1.7)	–
[42]	144 (100)	–	NR	NR	NR	NR	NR	NR	NR	NR	NR	NR	NR
[44]	250 (100)	250	187 (74.8)	57 (22.8)	–	–	–	–	–	–	–	6 (2.4)	–
[47]	307 (100)	307	230 (74.9)	69 (22.5)	2 (0.7)	–	–	–	–	–	–	6 (2.0)	–

*NR*, not reported; a: studies were not explicit about whether a participant could report more than one race/ethnicity; b: studies differed on using “Black” or “AA” or “Black/AA”; c: studies differed in defining “other” (see Table 2); d: these patients were “ > 1 race.” This table provides the race and/or ethnicity demographic characteristics of the study subjects with obesity involved in each study presented in this systematic literature review

Five studies transparently reported subject recruitment methods in enough detail to assess how patients were selected for the approach or referenced their parent study’s transparent methodology with the same subject population [34, 38, 44, 46, 47]. All studies except one had well-described inclusion and exclusion criteria [37]. Only two studies were explicit about the duration of the recruitment period in the context of the entire study period [38, 48]. Four studies had a high risk of randomization bias due to being open-label or the design of the study prevented randomization [36, 38, 44, 48]. One study only discussed randomizing males with no mention of the randomization process for females; this study enrolled both sexes [41]. No study was clear about whether their included study site(s) enrolled representative subjects or had a representative research staff of usual site demographics for patient or care staff population for the relevant disease of obesity during the study period.

## Discussion

The aim of this systematic review was to evaluate existing racial/ethnic and gender diversity among the participants of clinical trials focused on obesity and its metabolic sequelae in diet, drug, and surgical interventions with published results. Our results indicate that a relative lack of diversity among clinical trial participants continues, with the majority of participants in the included trials on average reported as White non-Hispanic. This is an important finding given that approximately 49.9% of Black Americans and 45.6% of Hispanics are obese [49], yet these populations are underrepresented in obesity trials. Studies included in this review did not include the wide array of gender identities possible, focusing solely on biological sex as options for selection, with females representing the overwhelming majority of participants in most studies. To our knowledge, this is the first systematic review evaluating both the racial/ethnic and gender diversity of clinical trial participants in clinical trials focused on the management of obesity and its comorbid conditions in diet, drug, and surgical interventions.

The majority of prior studies examining the diversity of participants in clinical trials have been in the oncology and cardiology literature, noting continued stagnation of meaningful changes in representation despite the mandate for the increased call to action by the NIH Revitalization Act of 1993 [4–6, 8, 10, 11, 16]. In the literature focused on obesity, very few clinical trials have addressed the issue of diversity. The review by Pagoto and colleagues noted that behavioral weight loss trials do not recruit a representative population of men, especially those from minority backgrounds [50]. In this review, no studies focused on men, and only two studies had a racial/ethnic minority population as the majority of study participants.

During the final review of articles that met inclusion criteria, not all studies reported demographics for their participants, and for those that did, the selection of reporting categories such as “other” were not clearly defined. This is a pervasive issue despite guidelines by the International Committee of Medical Journal Editors and NIH, which defines the standards for the classification of federal data on race and ethnicity [51–56]. The editors of the *Journal of the American Medical Association* recently published “Updated Guidance on the Reporting of Race and Ethnicity in Medical and Science Journals” in response to ongoing issues with reporting, with specific recommendations on the correct use of terms such as sex and gender, and that authors should “define how they determined race or ethnicity and justify their relevance.” [57] Increased adherence to these guidelines when publishing clinical trial data is critically important to be fully transparent about the demographics of study participants.

While the prevalence of obesity among adults in the USA is highest in the southeast region [58], most of the states participating in the included clinical trials were located in regions of the country where the prevalence of obesity is lower. This may be the result of differences in NIH funding awarded to medical schools, as historically the majority of the top 10 institutions in this regard have not been located in the southeast [59]. In recent years, this trend has continued.

With noted geographical differences in trial sites, an additional consideration is differences in sites located in urban versus rural locations, as rurality is a known factor to negatively impact access to care and overall health [60, 61]. As a result, study sites located in urban environments can overlook populations that would most benefit from participation in these clinical trials.

One method to increase diversity in clinical trials would be to address how obesity is defined. Obesity is traditionally categorized as starting at a body mass index (BMI) > 30 by both the World Health Organization and the Centers for Disease Control [62, 63]. This BMI cutoff is not inclusive of the effects of obesity for all racial/ethnic populations. Given the metabolic effects observed on Asian populations at lower BMIs, the Asian-Pacific recommendations are that BMI categories should be lowered in this population as follows: 23–24.9 for overweight and obese ≥ 25. For consideration of eligibility for bariatric and metabolic surgery, historically, the 1991 National Institutes of Health guidelines have been used since their release. These guidelines specify eligibility as a BMI ≥ 35 with obesity-related comorbid conditions or BMI > 40 despite endorsements by the American Diabetes Association [64] for lower BMI ranges for Americans (BMI ≥ 30) and Asians with Type 2 diabetes (BMI ≥ 27.5) to undergo bariatric and metabolic surgery if their hyperglycemia is not adequately controlled despite optimal medical management. With the use of the traditional guidelines, many trials omitted the recruitment of participants of Asian descent.

The American Society for Metabolic and Bariatric Surgery (ASMBS) and the International Federation for Surgery of Obesity and Metabolic Disorders (IFSO) [65] recently released new guidelines focused on the indications for bariatric and metabolic surgery, which include lower thresholds for both the Asian population similar to the Asian-Pacific recommendations and general population. With the recent joint release of the updated guidelines from ASMBS and IFSO, best practices would include the recommended lower BMI threshold of ≥ 30 for the general population for consideration of bariatric and metabolic surgery and ≥ 25 for those of Asian descent. It will be interesting to see if these guideline changes affect the diversity of participants in future clinical trials.

The findings of this review must be interpreted in the context of its limitations. One such limitation is the heterogeneity of reported race/ethnicity among the included studies, with 3 of the 18 studies not reporting this information as part of their cohort demographics, 7 studies reporting Hispanic ethnicity for participants, and only 5 studies reporting participants from Asian or Alaska Native/Native American populations. Another limitation is the heterogeneity of study types included, as we aimed to include not only diet-based and surgical weight-loss clinical trials for the treatment of obesity but also clinical trials with this patient population that address the common comorbid conditions associated with obesity. Clinicaltrial.gov registration pages do not consistently align with publications related to those registrations, as previously noted by Ludwig and colleagues [66]. In the case of our review, this inconsistency prohibited the verification of states/centers participating in the clinical trials for several studies. Articles included in this review were not restricted to a specific period of time, which may have excluded early obesity trials conducted before phases 3 and 4 trials were categorized. Trials with non-published data were not included in this review due to concerns regarding transparency and accuracy of demographic reporting. Lastly, as very few studies commented on participant enrollment methodology (including the timeframe over which they were recruited), there is a high risk of selection bias in the enrollment process for the included trials, raising the possibility of the influence of implicit bias as a confounder in any attempt at diversification of participants.

## Conclusion

Diverse representation of underrepresented groups and the disparities in reporting of race/ethnicity and gender in clinical trials remains pervasive issue. Future work in clinical trials focused on the treatment of obesity and its associated comorbid conditions should focus on the intentional recruiting of underrepresented populations to reflect the populations’ demographic.

### Supplementary Information

Below is the link to the electronic supplementary material.

Supplementary file1 (DOCX 39 KB)

## Data Availability

Detailed search methods are provided in the Supplemental Information. Further information may be available upon request from the author.

## Materials Availability

Detailed search methods are provided in the Supplemental Information. Further information may be available upon request from the author.

## References

[1] Hales CM (2018). Trends in obesity and severe obesity prevalence in US youth and adults by sex and age, 2007–2008 to 2015–2016. JAMA.

[2] Hales CM (2020). Prevalence of obesity and severe obesity among adults: United States, 2017–2018. NCHS Data Brief.

[3] United States Census Bureau. *QuickFacts*. 2021 2021 July 1 [cited 2022 March 1]; Available from: https://www.census.gov/quickfacts/fact/table/US/PST045221.

[4] Duma N (2018). Representation of minorities and women in oncology clinical trials: review of the past 14 years. J Oncol Pract.

[5] Sullivan LT (2018). Representation of black patients in randomized clinical trials of heart failure with reduced ejection fraction. Am Heart J.

[6] Chen MS Jr, et al. Twenty years post-NIH Revitalization Act: enhancing minority participation in clinical trials (EMPaCT): laying the groundwork for improving minority clinical trial accrual: renewing the case for enhancing minority participation in cancer clinical trials. Cancer, 2014. 120 Suppl 7(0 7): 1091–6. 10.1002/cncr.28575 PMC3980490 24643646

[7] Niranjan SJ (2020). Bias and stereotyping among research and clinical professionals: perspectives on minority recruitment for oncology clinical trials. Cancer.

[8] Tahhan AS (2020). Enrollment of older patients, women, and racial/ethnic minority groups in contemporary acute coronary syndrome clinical trials: a systematic review. JAMA Cardiol.

[9] Parekh T, Desai A (2022). Demographic and socioeconomic disparities among cancer survivors in clinical trials participation, USA, 2016–2018. J Cancer Educ.

[10] Unger JM (2020). Representativeness of black patients in cancer clinical trials sponsored by the National Cancer Institute compared with pharmaceutical companies. JNCI Cancer Spectr.

[11] Khan MS (2020). Ten-year trends in enrollment of women and minorities in pivotal trials supporting recent US Food and Drug Administration approval of novel cardiometabolic drugs. J Am Heart Assoc.

[12] Oh SS (2015). Diversity in clinical and biomedical research: a promise yet to be fulfilled. PLoS Med.

[13] Mishriky BM (2019). Do GLP-1RAs and SGLT-2is reduce cardiovascular events in black patients with type 2 diabetes? A systematic review and meta-analysis. Diabetes Obes Metab.

[14] Clark LT (2019). Increasing diversity in clinical trials: overcoming critical barriers. Curr Probl Cardiol.

[15] Hamel LM (2016). Barriers to clinical trial enrollment in racial and ethnic minority patients with cancer. Cancer Control.

[16] *National Institutes of Health Revitalization Act of 1993, 103rd United States Congress*. June 10 1993.

[17] Chang MW, Brown R, Nitzke S (2009). Participant recruitment and retention in a pilot program to prevent weight gain in low-income overweight and obese mothers. BMC Public Health.

[18] Brown SD (2012). Minority recruitment into clinical trials: experimental findings and practical implications. Contemp Clin Trials.

[19] Bennett GG (2013). Behavioral treatment for weight gain prevention among black women in primary care practice: a randomized clinical trial. JAMA Intern Med.

[20] Rosas LG (2015). The effectiveness of two community-based weight loss strategies among obese, low-income US Latinos. J Acad Nutr Diet.

[21] Turk MW (2009). Randomized clinical trials of weight loss maintenance: a review. J Cardiovasc Nurs.

[22] Khoo TK, Lin J (2021). Once-weekly semaglutide in adults with overweight or obesity. N Engl J Med.

[23] Rubino D (2021). Effect of continued weekly subcutaneous semaglutide vs placebo on weight loss maintenance in adults with overweight or obesity: the STEP 4 randomized clinical trial. JAMA.

[24] Campos GM (2020). Changes in utilization of bariatric surgery in the United States from 1993 to 2016. Ann Surg.

[25] Adams TD, Davidson LE, Hunt SC (2018). Weight and metabolic outcomes 12 years after gastric bypass. N Engl J Med.

[26] Courcoulas AP (2018). Seven-year weight trajectories and health outcomes in the Longitudinal Assessment of Bariatric Surgery (LABS) study. JAMA Surg.

[27] Page MJ (2021). The PRISMA 2020 statement: an updated guideline for reporting systematic reviews. Syst Rev.

[28] Higgins JP (2011). The Cochrane Collaboration’s tool for assessing risk of bias in randomised trials. BMJ.

[29] Sterne JAC (2019). RoB 2: a revised tool for assessing risk of bias in randomised trials. BMJ.

[30] Downs SH, Black N (1998). The feasibility of creating a checklist for the assessment of the methodological quality both of randomised and non-randomised studies of health care interventions. J Epidemiol Community Health.

[31] Aronne LJ (2013). Evaluation of phentermine and topiramate versus phentermine/topiramate extended-release in obese adults. Obesity (Silver Spring).

[32] Basolo A (2018). Exenatide has a pronounced effect on energy intake but not energy expenditure in non-diabetic subjects with obesity: a randomized, double-blind, placebo-controlled trial. Metabolism.

[33] Mende CW (2017). Efficacy of nebivolol-valsartan single-pill combination in obese and nonobese patients with hypertension. J Clin Hypertens (Greenwich).

[34] Acosta A et al. Exenatide in obesity with accelerated gastric emptying: a randomized, pharmacodynamics study. Physiol Rep, 2015. 3(11). 10.14814/phy2.12610 PMC4632965 26542264

[35] Acosta A (2015). Quantitative gastrointestinal and psychological traits associated with obesity and response to weight-loss therapy. Gastroenterology.

[36] Hsueh WA (2012). Efficacy of amlodipine/olmesartan medoxomil ± HCTZ in obese patients uncontrolled on antihypertensive monotherapy. Curr Med Res Opin.

[37] Koska J (2009). The effect of salsalate on insulin action and glucose tolerance in obese non-diabetic patients: results of a randomised double-blind placebo-controlled study. Diabetologia.

[38] Malone M, Alger-Mayer SA, Lindstrom J (2012). Use of Orlistat 60 mg in the management of weight loss before bariatric surgery. Ann Pharmacother.

[39] Neeland IJ (2020). Effects of empagliflozin treatment on glycerol-derived hepatic gluconeogenesis in adults with obesity: a randomized clinical trial. Obesity (Silver Spring).

[40] Perlstein TS (2012). Effect of angiotensin receptor blockade on insulin sensitivity and endothelial function in abdominally obese hypertensive patients with impaired fasting glucose. Clin Sci (Lond).

[41] Iqbal N (2010). Effects of a low-intensity intervention that prescribed a low-carbohydrate vs. a low-fat diet in obese, diabetic participants. Obesity (Silver Spring).

[42] Niswender KD (2018). Balanced high fat diet reduces cardiovascular risk in obese women although changes in adipose tissue, lipoproteins, and insulin resistance differ by race. Metabolism.

[43] Silver HJ (2014). Consuming a balanced high fat diet for 16 weeks improves body composition, inflammation and vascular function parameters in obese premenopausal women. Metabolism.

[44] Borradaile KE (2012). Relationship between treatment preference and weight loss in the context of a randomized controlled trial. Obesity (Silver Spring).

[45] de las Fuentes L (2009). Effect of moderate diet-induced weight loss and weight regain on cardiovascular structure and function. J Am Coll Cardiol..

[46] Foster GD (2010). Weight and metabolic outcomes after 2 years on a low-carbohydrate versus low-fat diet: a randomized trial. Ann Intern Med.

[47] Friedman AN (2012). Comparative effects of low-carbohydrate high-protein versus low-fat diets on the kidney. Clin J Am Soc Nephrol.

[48] Phillips E (2009). Safety and effectiveness of realize adjustable gastric band: 3-year prospective study in the United States. Surg Obes Relat Dis.

[49] *National Health and Nutrition Examination Survey 2017–March 2020 prepandemic data files development of files and prevalence estimates for selected health outcomes*, in *National Health Statistics Reports*, S. National Center for Health, Editor. 2021, 10.15620/cdc:106273: Hyattsville, MD. 10.15620/cdc:106273 PMC11513744 39380201

[50] Pagoto SL (2012). Male inclusion in randomized controlled trials of lifestyle weight loss interventions. Obesity (Silver Spring).

[51] Maduka RC (2021). The reporting of race and ethnicity in surgery literature. JAMA Surg.

[52] Loree JM (2019). Disparity of race reporting and representation in clinical trials leading to cancer drug approvals from 2008 to 2018. JAMA Oncol.

[53] Bokor-Billmann T, Langan EA, Billmann F (2020). The reporting of race and/or ethnicity in the medical literature: a retrospective bibliometric analysis confirmed room for improvement. J Clin Epidemiol.

[54] International Committee of Medical Journal Editors. *Recommendations for the conduct, reporting, editing, and publication of scholarly work in medical journals*. 2021 2021 December [cited 2021 November 18]; Available from: https://www.icmje.org/recommendations/. 25558501

[55] *National Institutes of Health Notice - racial and ethnic categories and definitions for NIH diversity programs and for other reporting purposes*. April 8 2015.

[56] *United States Office of Management and Budget, federal register notice - revisions to the standards for the classification of federal data on race and ethnicity*. October 30 1997.

[57] Flanagin A, Frey T, Christiansen SL (2021). Updated guidance on the reporting of race and ethnicity in medical and science journals. JAMA.

[58] Centers for Disease Control and Prevention. *Adult obesity prevalence maps*. 2021 2021 September 27 [cited 2022 March 15]; Available from: https://www.cdc.gov/obesity/data/prevalence-maps.html#overall.

[59] Parslow, T.G. and R. Roskoski Jr. *Ranking tables of NIH funding to US medical schools*. 2022 [cited 2022 March 15]; Available from: http://www.brimr.org/NIH_Awards/NIH_Awards.htm.

[60] Garcia MC (2017). Reducing potentially excess deaths from the five leading causes of death in the rural United States. MMWR Surveill Summ.

[61] University of North Carolina Cecil G. Sheps Center for Health Services Research. *Rural hospital closures 2005-current*. 2022 [cited 2022 March 15]; Available from: https://www.shepscenter.unc.edu/programs-projects/rural-health/rural-hospital-closures/.

[62] World Health Organization. *Obesity and overweight*. 2021 [cited 2022 2 November 2022]; Available from: https://www.who.int/news-room/fact-sheets/detail/obesity-and-overweight#:~:text=%2Fm2).-,Adults,than%20or%20equal%20to%2030.

[63] Centers for Disease Control and Prevention. *Defining adult overweight & obesity*. 2022 [cited 2022 2 November 2022]; Available from: https://www.cdc.gov/obesity/basics/adult-defining.html.

[64] 7. Obesity management for the treatment of type 2 diabetes: standards of medical care in diabetes-2018. Diabetes Care, 2018. 41(Suppl 1): S65-s72. 10.2337/dc18-S007 29222378

[65] Eisenberg, D., et al., *2022 American Society for Metabolic and Bariatric Surgery (ASMBS) and International Federation for the Surgery of Obesity and Metabolic Disorders (IFSO): indications for metabolic and bariatric surgery.* Surg Obes Relat Dis, 2022. 10.1016/j.soard.2022.08.013 36280539

[66] Ludwig DS, Ebbeling CB, Heymsfield SB (2019). Discrepancies in the registries of diet vs drug trials. JAMA Netw Open.

