# Supplementary material for: A Systematic Review on Participant Diversity in Clinical Trials—Have We Made Progress for the Management of Obesity and Its Metabolic Sequelae in Diet, Drug, and Surgical Trials
Source: J Racial Ethn Health Disparities. 2022 Dec 19;10(6):3140–9. doi: 10.1007/s40615-022-01487-0 (PMC10645628; doi:10.1007/s40615-022-01487-0)
Supplement: Supplementary file 1 — Supplementary file1 (DOCX 39 KB) [file 40615_2022_1487_MOESM1_ESM.docx]

**Supporting Information**

**A systematic review on participant diversity in clinical trials – have we made progress for the management of obesity and its metabolic sequelae in diet, drug, and surgical trials?**

Crystal N. Johnson-Mann MD MPH^1,2^, Julie S. Cupka BS^1,3^, Alexandra Ro BA^1,3^, Andrea E. Davidson BS^1,3^, Brooke A. Armfield PhD^1^, Frank Miralles BS^4^, Asena Markal BS^4^, Kiara Fierman^1,3^, Victoria Hough^1,3^, Mackenzie Newsom^1,3^, Isha Verma^1,3^, Abdul-Vehab Dozic^1,3^, Azra Bihorac MD MS^1,3^

1. Intelligent Critical Care Center, University of Florida, Gainesville, Florida, USA
2. Division of Gastrointestinal Surgery, Department of Surgery, College of Medicine, University of Florida, Gainesville, Florida, USA
3. Division of Nephrology, Hypertension and Renal Transplantation, Department of Medicine, College of Medicine, University of Florida, Gainesville, Florida, USA
4. College of Medicine, University of Florida, Gainesville, Florida, USA

**Contact info:**

Crystal N. Johnson-Mann, MD MPH

1600 SW Archer Rd

PO BOX 100109

Gainesville, Florida 32610-0109

[Crystal.Johnson-Mann@surgery.ufl.edu](mailto:Crystal.Johnson-Mann@surgery.ufl.edu)

Table of Contents

Supplemental Appendix 1: PRISMA 2020 Checklist

Supplemental Appendix 2: Search Terms and Strategies

Supplemental Appendix 3: Risk of Bias Worksheet

| **Section and Topic** | **Item #** | **Checklist item** | **Location where item is reported** |
| --- | --- | --- | --- |
| **TITLE** | | |  |
| Title | 1 | Identify the report as a systematic review. | Pg 1 |
| **ABSTRACT** | | |  |
| Abstract | 2 | See the PRISMA 2020 for Abstracts checklist. | Pg 3 |
| **INTRODUCTION** | | |  |
| Rationale | 3 | Describe the rationale for the review in the context of existing knowledge. | Pg 4-5 |
| Objectives | 4 | Provide an explicit statement of the objective(s) or question(s) the review addresses. | Pg 5 |
| **METHODS** | | |  |
| Eligibility criteria | 5 | Specify the inclusion and exclusion criteria for the review and how studies were grouped for the syntheses. | Pg 5-6 |
| Information sources | 6 | Specify all databases, registers, websites, organisations, reference lists and other sources searched or consulted to identify studies. Specify the date when each source was last searched or consulted. | Pg 5 |
| Search strategy | 7 | Present the full search strategies for all databases, registers and websites, including any filters and limits used. | Pg 5-6, Appendix S2 |
| Selection process | 8 | Specify the methods used to decide whether a study met the inclusion criteria of the review, including how many reviewers screened each record and each report retrieved, whether they worked independently, and if applicable, details of automation tools used in the process. | Pg 5-6 |
| Data collection process | 9 | Specify the methods used to collect data from reports, including how many reviewers collected data from each report, whether they worked independently, any processes for obtaining or confirming data from study investigators, and if applicable, details of automation tools used in the process. | Pg 5-6 |
| Data items | 10a | List and define all outcomes for which data were sought. Specify whether all results that were compatible with each outcome domain in each study were sought (e.g. for all measures, time points, analyses), and if not, the methods used to decide which results to collect. | Pg 5-6 |
|  | 10b | List and define all other variables for which data were sought (e.g. participant and intervention characteristics, funding sources). Describe any assumptions made about any missing or unclear information. | Pg 5-6 |
| Study risk of bias assessment | 11 | Specify the methods used to assess risk of bias in the included studies, including details of the tool(s) used, how many reviewers assessed each study and whether they worked independently, and if applicable, details of automation tools used in the process. | Pg 6-7; Appendix S3 |
| Effect measures | 12 | Specify for each outcome the effect measure(s) (e.g. risk ratio, mean difference) used in the synthesis or presentation of results. | n/a – demographic report |
| Synthesis methods | 13a | Describe the processes used to decide which studies were eligible for each synthesis (e.g. tabulating the study intervention characteristics and comparing against the planned groups for each synthesis (item #5)). | n/a – demographic report |
|  | 13b | Describe any methods required to prepare the data for presentation or synthesis, such as handling of missing summary statistics, or data conversions. | n/a – demographic report |
|  | 13c | Describe any methods used to tabulate or visually display results of individual studies and syntheses. | Table 1-3; Figure 2 |
|  | 13d | Describe any methods used to synthesize results and provide a rationale for the choice(s). If meta-analysis was performed, describe the model(s), method(s) to identify the presence and extent of statistical heterogeneity, and software package(s) used. | n/a – demographic report |
|  | 13e | Describe any methods used to explore possible causes of heterogeneity among study results (e.g. subgroup analysis, meta-regression). | n/a – demographic report |
|  | 13f | Describe any sensitivity analyses conducted to assess robustness of the synthesized results. | n/a – demographic report |
| Reporting bias assessment | 14 | Describe any methods used to assess risk of bias due to missing results in a synthesis (arising from reporting biases). | Pg 5-6 |
| Certainty assessment | 15 | Describe any methods used to assess certainty (or confidence) in the body of evidence for an outcome. | n/a – demographic report |
| **RESULTS** | | |  |
| Study selection | 16a | Describe the results of the search and selection process, from the number of records identified in the search to the number of studies included in the review, ideally using a flow diagram. | Pg 5 & 7; Figure 1 |
|  | 16b | Cite studies that might appear to meet the inclusion criteria, but which were excluded, and explain why they were excluded. | n/a |
| Study characteristics | 17 | Cite each included study and present its characteristics. | Pg 7-8; Tables 1-3; Figure 2 |
| Risk of bias in studies | 18 | Present assessments of risk of bias for each included study. | Pg 8 |
| Results of individual studies | 19 | For all outcomes, present, for each study: (a) summary statistics for each group (where appropriate) and (b) an effect estimate and its precision (e.g. confidence/credible interval), ideally using structured tables or plots. | Pg 7-8; Tables 1-3; Figure 2 |
| Results of syntheses | 20a | For each synthesis, briefly summarise the characteristics and risk of bias among contributing studies. | Pg 8 |
|  | 20b | Present results of all statistical syntheses conducted. If meta-analysis was done, present for each the summary estimate and its precision (e.g. confidence/credible interval) and measures of statistical heterogeneity. If comparing groups, describe the direction of the effect. | n/a – demographic report |
|  | 20c | Present results of all investigations of possible causes of heterogeneity among study results. | n/a – demographic report |
|  | 20d | Present results of all sensitivity analyses conducted to assess the robustness of the synthesized results. | n/a – demographic report |
| Reporting biases | 21 | Present assessments of risk of bias due to missing results (arising from reporting biases) for each synthesis assessed. | Pg 8 |
| Certainty of evidence | 22 | Present assessments of certainty (or confidence) in the body of evidence for each outcome assessed. | n/a – demographic report |
| **DISCUSSION** | | |  |
| Discussion | 23a | Provide a general interpretation of the results in the context of other evidence. | Pg 8-10 |
|  | 23b | Discuss any limitations of the evidence included in the review. | Pg 8-10 |
|  | 23c | Discuss any limitations of the review processes used. | Pg 9-11 |
|  | 23d | Discuss implications of the results for practice, policy, and future research. | Pg 8-11 |
| **OTHER INFORMATION** | | |  |
| Registration and protocol | 24a | Provide registration information for the review, including register name and registration number, or state that the review was not registered. | Pg 6 |
|  | 24b | Indicate where the review protocol can be accessed, or state that a protocol was not prepared. | Pg 6 |
|  | 24c | Describe and explain any amendments to information provided at registration or in the protocol. | n/a |
| Support | 25 | Describe sources of financial or non-financial support for the review, and the role of the funders or sponsors in the review. | Pg 1 |
| Competing interests | 26 | Declare any competing interests of review authors. | Pg 1 |
| Availability of data, code and other materials | 27 | Report which of the following are publicly available and where they can be found: template data collection forms; data extracted from included studies; data used for all analyses; analytic code; any other materials used in the review. | Pg 1 |

*From:*  Page MJ, McKenzie JE, Bossuyt PM, Boutron I, Hoffmann TC, Mulrow CD, et al. The PRISMA 2020 statement: an updated guideline for reporting systematic reviews. BMJ 2021;372:n71. doi: 10.1136/bmj.n71

For more information, visit: <http://www.prisma-statement.org/>

Supplemental Appendix 2: Search Terms and Strategies.

**PUBMED**

("Obesity"[Mesh] OR obes*[tiab])

AND

("Clinical Trial, Phase III"[Publication Type] OR "Clinical Trial, Phase IV"[Publication Type] OR “phase 3”[tw] OR “phase III”[tw] OR “phase 4”[tw] OR “phase IV”[tw])

**COCHRANE**

(MeSH:obesity OR (All Text:obes*))

AND

(All Text: ((phase III) or (phase 3) or (phase IV) or (phase 4)))

**WEB OF SCIENCE**

TS=(obes*)

AND

ALL=(“phase 3” OR “phase III” OR “phase 4” OR “phase IV”)

**CLINICALTRIALS.GOV**

1. Go to ‘Advanced Search’
2. Fill the following fields
   1. Condition or disease: Obesity
   2. Study type: Interventional Studies (Clinical Trials)
   3. Recruitment: Completed
   4. Country: United States
   5. Phase: Phase 3; Phase 4
3. Click ‘Search’
4. Export results

**Supplemental Appendix 3: Risk of Bias Worksheet**

| Study: |
| --- |

| Randomization & Allocation | Was the allocation random? | Comments: | Verdict:  Yes / No / Not Enough Available Information |
| --- | --- | --- | --- |
|  | Was the allocation sequence concealed until participants were enrolled and assigned to interventions? | Comments: | Verdict:  Yes / No / Not Enough Available Information |
|  | Did baseline differences between intervention groups suggest a problem with the randomization process? | Comments: | Verdict:  Yes / No / Not Enough Available Information |
|  | Overall judgement | Comments: | Verdict:  Low risk / High risk / Have Concerns |

| Recruitment & Participant Details | **Are the characteristics of the patients included in the study clearly described?** **Reporting Domain.** In cohort studies and trials, inclusion and/or exclusion criteria should be given. In case-control studies, a case-definition and the source for controls should be given. | Comments: | Verdict:  Yes / Partial / No |
| --- | --- | --- | --- |
|  | **Are the distributions of principal confounders in each group of subjects to be compared clearly described?** **Reporting Domain.** A list of principal confounders is provided by the study. | Comments: | Verdict:  Yes / Partial / No |
|  | **Were losses of patients to follow-up taken into account? Internal Validity (confounding; selection bias) Domain.** If the numbers of patients lost to follow-up are not reported, the question should be answered as ‘unable to determine’ If the proportion lost to follow-up was too small to affect the main findings, the question should be answered yes. | Comments: | Verdict:  Yes / No / Unable to Determine |
|  | **Have the characteristics of patients lost to follow-up been described?** **Reporting Domain.** This should be answered ‘yes’ where there were no losses to follow-up or where losses to follow-up were so small that findings would be unaffected by their inclusion. This should be answered ‘no’ where a study does no report the number of patients lost to follow-up. | Comments: | Verdict:  Yes / Partial / No |
|  | **Were the subjects asked to participate in the study representative of the entire population from which they were recruited?** **External Validity Domain.** The study must identify the source population for patients and describe how the patients were selected. Patients would be representative if they comprised the entire source population, an unselected sample of consecutive patients, or a random sample. Random sampling is only feasible where a list of all members of the relevant population exists. Where a study does not report the proportion of the source population from which the patients are derived, the question should be answered as ‘unable to determine’. | Comments: | Verdict:  Yes / No / Unable to Determine |
|  | **Were those subjects who were prepared to participate representative of the entire population from which they were recruited?** **External Validity Domain.** The proportion of those asked who agreed should be stated. Validation that the sample was representative would include demonstrating that the distribution of the main confounding factors was the same in the study sample and the source population. | Comments: | Verdict:  Yes / No / Unable to Determine |
|  | **Were the staff, places, and facilities where the patients were treated representative of the treatment the majority of patients receive?** **External Validity Domain.** For the question to be answered yes the study should demonstrate that the intervention was representative of that in use in the source population. The question should be answered no if, for example, the intervention was undertaken in a specialist centre unrepresentative of the hospitals most of the source population would attend. | Comments: | Verdict:  Yes / No / Unable to Determine |
|  | **Were the patients in different intervention groups (trials and cohort studies) or were the cases and controls (case-control studies) recruited from the same population?** **Internal Validity (confounding; selection bias) Domain.** For example, patients for all comparison groups should be selected from the same hospital. The question should be answered ‘unable to determine’ for cohort and case-control studies were there is no information concerning the source of patients included in the study. | Comments: | Verdict:  Yes / No / Unable to Determine |
|  | **Were study subjects in different intervention groups (trials and cohort studies) or were the cases and controls (case-control studies) recruited over the same period of time?** **Internal Validity (confounding; selection bias) Domain.** For a study which does not specify the time period over which patients were recruited, the question should be answered as ‘unable to determine’. | Comments: | Verdict:  Yes / No / Unable to Determine |
